# Surgical Outcomes and Prognostic Factors of Myxoid Liposarcoma in Extremities: A Retrospective Study

**DOI:** 10.1111/os.12566

**Published:** 2019-11-12

**Authors:** Kai Zheng, Xiu‐chun Yu, Ming Xu, Yang Yang

**Affiliations:** ^1^ Department of Orthopaedics The 960th Hospital of the PLA Joint Logistice Support Force Jinan China

**Keywords:** Extremities, Myxoid liposarcoma, Prognostic factors, Surgical outcome

## Abstract

**Objectives:**

To evaluate treatments and prognostic factors for the myxoid liposarcoma in extremities.

**Methods:**

We retrospectively reviewed 34 patients histologically diagnosed with myxoid liposarcoma arising in the extremities, treated in our hospital from 2010 to 2017. We recorded tumor locations, max diameter, operations, complications, radiation, chemotherapy, survival, recrudescence, and metastases. Overall survival, treatments, and prognostic factors were subsequently analyzed.

**Results:**

The mean age of 34 patients with myxoid liposarcoma in extremities was 49.1 years, and the mean follow‐up period was 65.1 months. The median survival time was 190 months. Five of 14 patients accepted recrudescence resection and two patients of 20 patients who underwent primary tumor resection or unplanned operation died of tumor progression. Although no statistical difference was found (*X* ^*2*^ = 3.331, *P* = 0.068), the lower mortality was confirmed in the patients who accepted primary tumor resection or unplanned operation. Eleven patients with a tumor diameter of 8.6 ± 4.7 cm accepted wide resection, while 23 patients with 17.2 ± 8.8 cm tumors accepted marginal resection. Statistical difference was found between the size of tumors with relatively wide resection and those with relatively marginal resection (*F* = 9.130, *P* = 0.005). No recurrence or metastasis occurred in patients who accepted wide resection, while 14 patients presented with local recurrence and 8 patients developed distant metastases among the 23 patients with marginal resection. Seven patients died of metastases, while one patient lived with metastases. No significant difference in survival was found between different surgical methods (*X* ^*2*^ = 0.9460, *P* = 0.3307). The average diameter of eight patients with distant metastases was 21.7 cm, which was considerable larger than the 12.1 cm of patients without metastasis. This difference was proven significant upon the statistical analysis (*F *= 9.412, *P* = 0.004).

**Conclusions:**

Wide resection achieved good local control but was not unambiguously superior in long‐term survival. Myxoid liposarcoma tumors with larger diameters were more difficult to be submitted to wide resection and were more likely to present with distant metastasis.

## Introduction

Myxoid liposarcoma (MLPS) accounts for 15%–20% of liposarcomas and represents about 5% of all soft tissue sarcomas in adults^1^. Although liposarcoma can occur in any part of the body^2^, ^3^, MLPS occurs more commonly in the deep soft tissues of extremities, and in more than two thirds of cases it develops within the musculature of the thigh^1^, ^2^. More than 90% of MLPSs contain a pathognomonic t (12; 16) (q13; p11) translocation that results in the expression of the FUS‐DDIT3 fusion protein, whereas a smaller proportion carries the EWSR1‐DDIT3 gene fusion^3^, ^4^. Round cell liposarcoma is recognized as a high‐grade, cell‐specific variant of MLPS characterized by poor prognosis^2^, ^5^. Local recurrence and metastasis to atypical sites, such as bone, retroperitoneum, or lymph nodes, are also commonly encountered^2^, ^5^, ^6^, ^7^, ^8^, ^9^. The treatment options for soft tissue sarcoma consist of tumor resection, neoadjuvant chemotherapy, adjuvant chemotherapy, and radiotherapy^2^, ^10^, ^11^, ^12^, ^13^, ^14^, ^15^, ^16^. The roles of chemotherapy and radiotherapy in treating localized soft tissue sarcoma remain controversial. For most soft tissue sarcomas, tumor resection with a safe surgical margin is recommended, although perfect surgical margins are difficult to attain in sarcomas with large size or adjacent to important vessels and nerves. Although MLPS has greater chemosensitivity to adjuvant chemotherapy^12^, ^13^, ^17^, tumor resection is the first choice of treatment. At present, there is no consensus on the prognostic factors of MLPS^18^, ^19^, ^20^.

To date, few studies have investigated the clinical outcomes of MLPS in extremities. A retrospective study including 53 patients with MLPS in extremities and trunk wall reported that local control could be achieved by tumor resection with wide surgical margins, without radiotherapy, and age was an independent factor affecting patient prognosis, whereas the tumor size and depth were not^19^. Another retrospective study including 49 patients with the median follow‐up of 101 months reported that the age at presentation, tumor grade, and tumor size had a negative influence on survival, whereas the tumor grade was the only independent prognostic factor^18^. Additionally, a study on prognostic factors for patients with myxoid/round‐cell liposarcoma found that the occurrence of any round cell component is the most important adverse prognostic factor^20^.

Although some studies have reported on the treatment effects and prognostic factors of MLPS, the conclusions were inconsistent and sometimes contradictory. To reduce the number of confounding factors in this study, the tumor location was limited to the limbs and the pathological type was limited to MLPS. The aims of this study were to determine: (i) the most suitable treatment for MLPS arising in the extremities; (ii) whether the patients have good local control and long‐term survival with wide resection; and (iii) whether any clinical or treatment characteristics influence survival.

## Materials and Methods

### *Patients*

Thirty‐four patients with MLPS in extremities received treatment at The 960th Hospital of the PLA Joint Logistics Support Force from January 2010 to June 2017. Patient data were collected from patient records, surgical protocols, and histological and radiological findings. The follow‐up time was calculated after the initial operation. The last follow‐up was performed *via* follow‐up examination after surgery or telephone contact. Ethical approval was granted by the Institutional Ethical Committee of Jinan Military General Hospital, and patient consent was also obtained for this study. The research was carried out according to the principles set out in the Declaration of Helsinki (1964) and all subsequent revisions.

### *Inclusion and Exclusion Criteria*

The inclusion criteria were: (i) pathological diagnosis of MLPS was definite; (ii) MLPS involved the extremities, but not the pelvis; and (iii) MLPS accepted resection.

The exclusion criteria were: (i) metastasis during initial treatment in our hospital; (ii) incomplete clinical, radiographic, and pathologic records; and (iii) no standardized follow‐up data.

### *Tumor Diagnosis*

All patients accepted general assessment and local tumor examination before treatments. The tumor max diameter was measured by magnetic resonance imaging (MRI) and computed tomography (CT). The diagnosis of MLPS was established based on clinical and imaging data, and confirmed by needle biopsy or open biopsy before surgery, as well as by pathological examination after surgery. Specimens of previously excised liposarcoma were reviewed by a professional pathologist in our hospital.

### *Treatment Strategy*

Study participants were divided into three types: the first type referred to patients that accepted diagnosis and treatment in our hospital; the second type consisted of patients that accepted immediate, unplanned operation in our hospital after inappropriate surgery in other hospitals; the third type was patients that accepted recrudescence resection in our hospital.

In accordance with the classification system for musculoskeletal sarcoma surgical methods by Enneking *et al*.^21^, which is based on the operation notes and pathology reports, wide resection was defined as *en bloc* resection through the normal tissue within the compartment of origin of the tumor. Marginal resection was defined as *en bloc* resection through a marginal reactive zone.

The patient demographics, clinical presentation, history of previous excised tumors, surgical methods, and administration of radiotherapy or chemotherapy were recorded.

### *Oncological Outcome*

#### *Overall Survival*

Survival status was evaluated according to both local and distant tumor control. All patients were requested to be reexamined every month for half a year after surgery, every 3 months between 0.5–2 years, every 6 months between 2–5 years, and annually after 5 years. Local disease control and metastases were recorded. Local recurrence was suspected initially by evidence of new mass upon physical examination and imaging studies, and a biopsy was further performed to confirm the suspicion. Local recurrences were re‐excised surgically and thoroughly examined histologically for any changes.

#### *Tumor Metastasis*

Pulmonary metastasis, bone metastasis, retroperitoneal metastasis, and other metastasis had been recorded in this study. Pulmonary metastasis was confirmed by chest CT while retroperitoneal metastasis and bone metastasis were confirmed by MRI.

#### *Surgical Timing on Survival*

Study participants were divided into three types according to surgical timing in this series. The patients' survival in different types were analyzed.

#### *Surgical Methods on Survival*

Surgical methods for these MLPS included wide resection and marginal resection. The effects of different surgical methods on patient survival were analyzed.

#### *Adjuvant Treatment on Survival*

In this series, adjuvant treatments included adjuvant chemotherapy and adjuvant radiotherapy. The effects of different adjuvant treatments on patient survival were analyzed.

### *Complications*

The clinical complications, such as infection and delayed incision healing, were recorded. Oncological failure was not recorded as a complication.

### *Statistical Analysis*

In this study, the SPSS 13.0 (Chicago, IL, USA) statistical software was used for data analysis. All survival data were analyzed using the Kaplan‐Meier method. Multiple comparisons of specific values between different groups of result‐oriented indicators were performed. The method of analysis of variance and chi‐square test were used for comparisons, and the differences were considered statistically significant when *P* < 0.05.

## Results

### *General Results*

**Figure 1:**
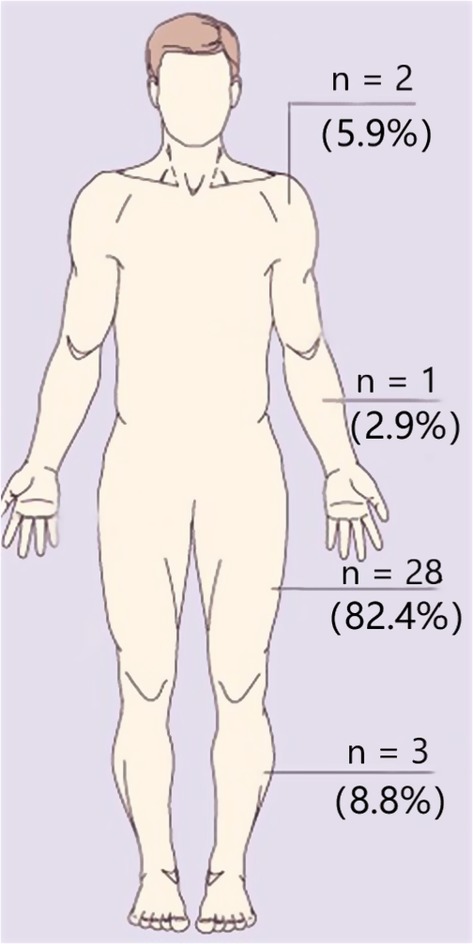
Tumor distribution of these 34 patients were showed. Three tumors were located on the upper extremities, including two in the shoulder and one in the arm, while 31 tumors were located on the lower extremities, including 28 in the thigh and three in the leg.

Clinical data for 34 patients with myxoid liposarcoma in extremities are summarized. There were 19 males and 15 females in this series, with the mean age of 49.1 years (range, 17–92 years). Three tumors were located on the upper extremities, including two in the shoulder and one in the arm, while 31 tumors were located on the lower extremities, including 28 in the thigh and three in the leg (Fig. 1). For tumors compartment distribution in the upper extremities, two tumors were located in unicompartment and one in multicompartment, while 24 tumors were located in unicompartment and seven in multicompartment in the lower extremities (Table 1).

**Table 1:** Compartment distribution of myxoid liposarcoma in 34 cases

	Unicompartment tumor	Multicompartment tumor
Upper extremities	2	1
Lower extremities	24	7

### *Treatment Outcome*

**Figure 2:**
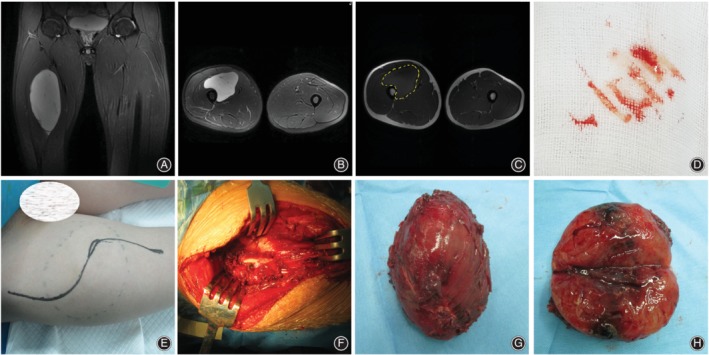
A 20‐year‐old male patient underwent primary myxoid liposarcoma resection in our hospital. (A) Coronal plane MRI showed tumor in the left thigh. (B) Cross plane MRI showed normal bone signal and clear tumor edge. (C) Yellow dotted line showed planned cut‐off boundary. (D) Puncture biopsy was recommended for preoperative diagnosis. (E) Puncture site was included in the surgical incision. (F) Periosteal resection was necessary. (G) The specimen boundary was tumor‐free under naked eyes. (H) Central necrosis could be found in the longitudinal sectioning of tumor.

**Figure 3:**
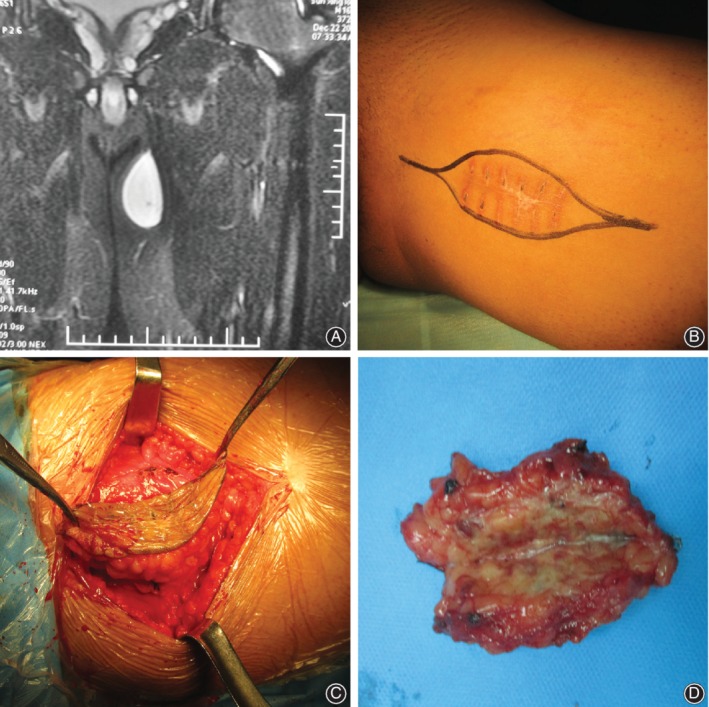
A sixteen‐year‐old male patient accepted unplanned operation immediately after inappropriate operations in other hospitals. (A) MRI before first operation showed soft tissue tumor with clear edge. (B) The reoperation boundary was outside of the initial surgical boundary. (C) The resection was carried in the normal tissue. (D) The specimen boundary should be tumor‐free under naked eyes.

**Figure 4:**
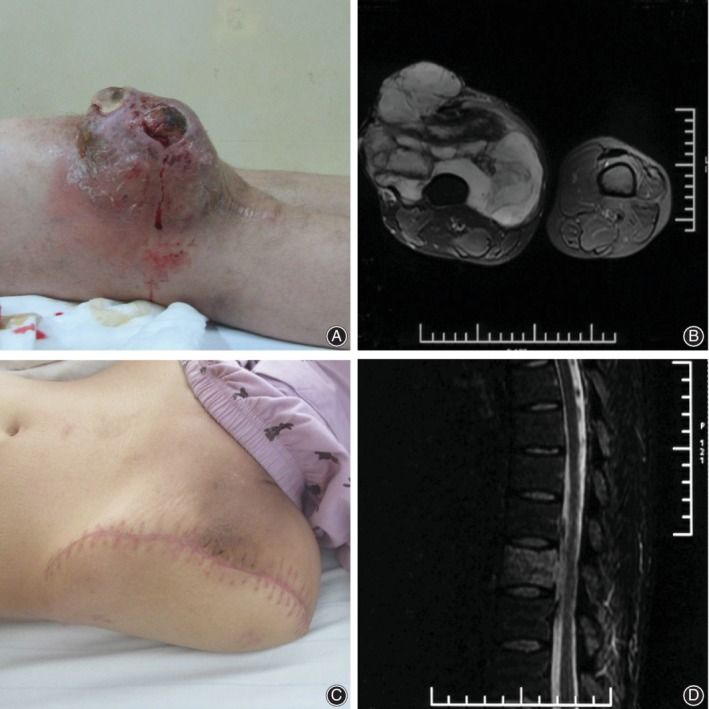
A twenty‐eight years old male patient underwent hip dissection because of myxoid liposarcoma recrudescence. (A) Myxoid liposarcoma recurred at one year after operation. (B) Cross plane MRI showed huge tumor (C) The patient accepted hip dissection. (D) MRI showed the thoracic metastasis of myxoid liposarcoma at three months after hip dissection.

These 34 patients underwent treatment in our hospital at three different disease stages. Specifically, 11 patients underwent primary tumor resection (Fig. 2), nine patients accepted unplanned operation immediately after inappropriate operations in other hospitals (Fig. 3), and 14 patients underwent recrudescence resection (Fig. 4). Limb‐salvaging surgery was the first choice for most patients, except for one patient in the unplanned operation group and four patients among the recurrence cases who accepted amputation.

In the evaluation of surgical approaches, 11 patients underwent wide resection and 23 patients accepted marginal resection. Tumor size was an important influencing factor when choosing the appropriate surgical method. The tumor diameter was 8.6 ± 4.7 cm in patients who accepted wide resection and 17.2 ± 8.8 cm in those who underwent marginal resection. There was a statistical difference in tumor size between these two groups (*F* = 9.130, *P* = 0.005).

### *Oncological Outcome*

#### *Overall Survival*

**Figure 5:**
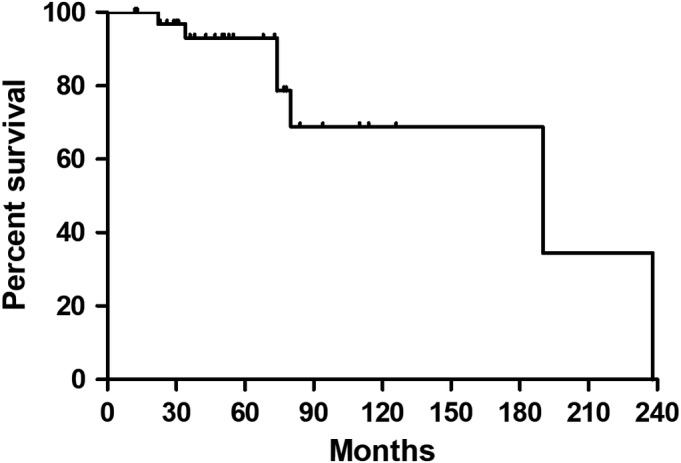
Survival curve of these 34 patients.

**Figure 6:**
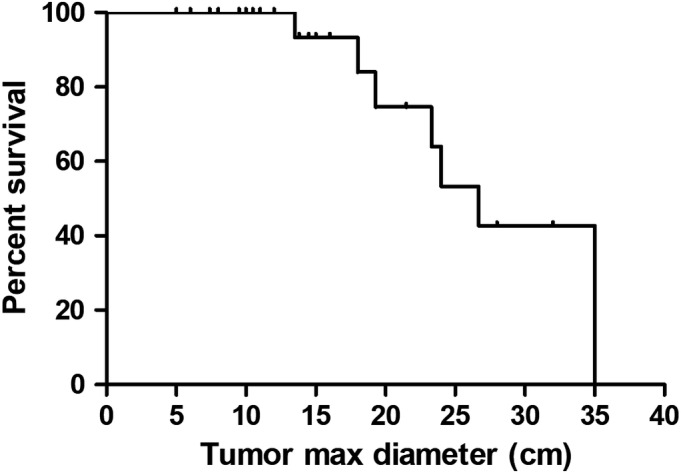
The effect of tumor diameter on survival was presented.

The mean follow‐up period was 65.1 months (range, 12–238 months). Seven patients (7/34, 20.6%) died of disease during the follow‐up. The median survival time was 190 months for this series (Fig. 5). The mean size of tumors, as measured by their maximum diameter, was 14.4 cm (range, 5–35 cm). The effect of tumor diameter on survival is presented in Fig. 6.

#### *Tumor Metastasis*

The half of the fourteen patients developed tumor recurrence developed disease‐free after repeated tumor resection while the other seven patients developed distant metastases. Only one patient developed distant metastases without local tumor recurrence. Distant metastases included three bone metastases, three lung metastases, one retroperitoneal metastasis, and one multiple metastasis. The average tumor size of eight patients with distant metastases was 21.7 cm compared to 12.1 cm of patients without metastasis, which was statistically significant (*F* = 9.412, *P* = 0.004).

#### *Surgical Timing on Survival*

**Figure 7:**
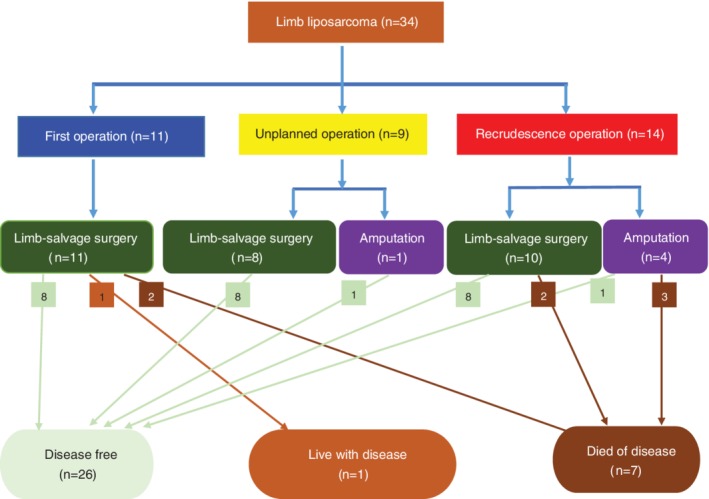
Surgical timing, surgical methods, and survival of these 34 patients

All patients in the unplanned operation group became disease‐free. Additionally, eight patients in the primary tumor resection group and nine patients in the recrudescence operation group became disease‐free, while two patients in the former group and five patients in the latter died of the disease, and one patient in the former group lived with the disease (Fig. 7). Patients who underwent recrudescence resection had higher mortality than patients in the primary tumor resection and unplanned operation groups, but the difference was not statistically significant (*X* ^*2*^ = 3.331, *P* = 0.068).

#### *Surgical Methods on Survival*

**Figure 8:**
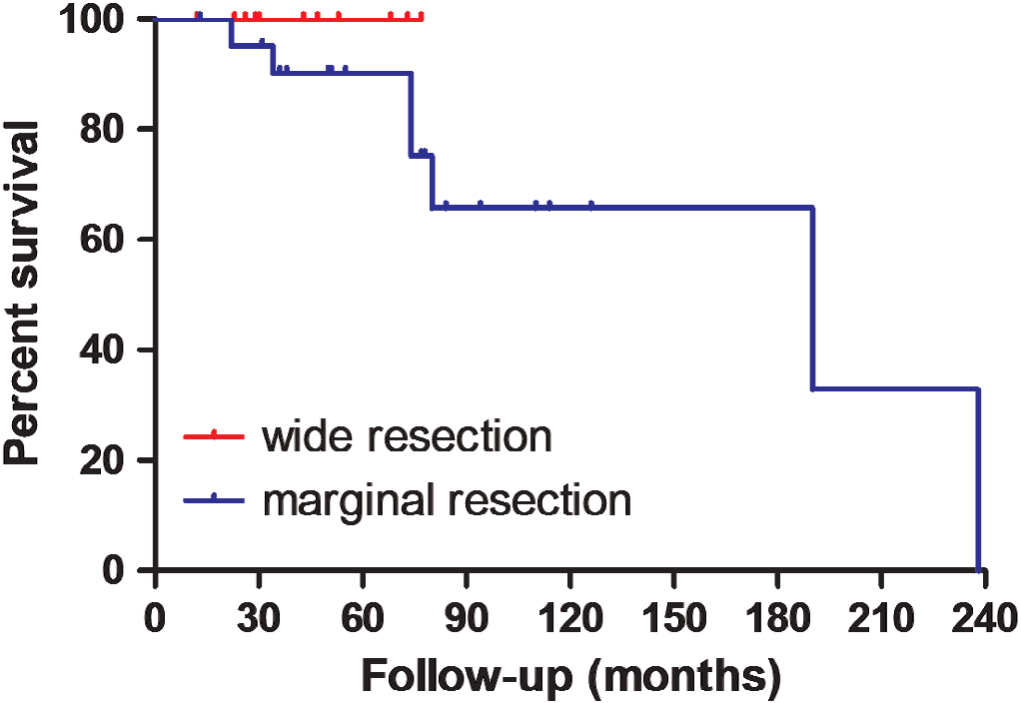
The effect of surgical methods on survival was presented.

No local recurrence or distant metastasis was found in patients who accepted wide resection during the follow‐up which averaged 43.7 months (12–77 months). Of 23 patients who underwent marginal resection, 14 (60.9%) developed local recurrence during the follow‐up period which averaged 75.3 months (13–238 months). Fourteen patients accepted repeated tumor resection at different times (from one to 11 repeat surgeries). Distant metastases occurred in 34.8% (8/23) of the patients. Of these, seven patients died of metastases, while one patient lived with metastasis. The survival curves are presented in Fig. 8, and no significant difference was found between the different surgical methods (*X* ^*2*^ = 0.9460, *P* = 0.3307).

#### *Adjuvant Treatment on Survival*

**Figure 9:**
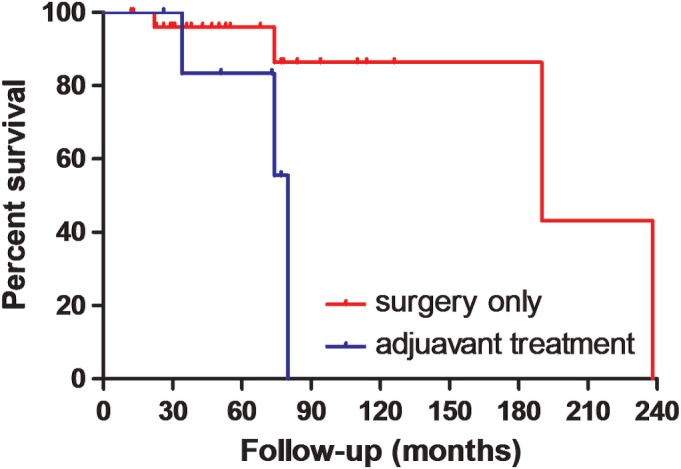
The effect of adjuvant treatment (chemotherapy or radiotherapy) on survival was presented.

Seven patients in this series accepted adjuvant chemotherapy or radiotherapy after tumor resection. Adjuvant chemotherapy consisted of doxorubicin and ifosfamide, while the dose of vitro radiotherapy was 30 Gy. Of these patients, two accepted adjuvant chemotherapy after wide resection and became disease‐free. After marginal resection, three patients accepted adjuvant chemotherapy and two patients accepted radiotherapy. Three patients died of the disease, while the other two patients lived disease‐free. The effect of adjuvant treatment (chemotherapy or radiotherapy) on survival is presented in Fig. 9. The median survival time was 190 months for patients who underwent surgery alone and 80 months for patients who accepted both surgery and adjuvant treatment. A significant difference was found upon statistical analysis (*X* ^*2*^ = 4.848, *P* = 0.028). The average tumor size of patients who accepted adjuvant treatments was 17.5 cm *versus* 13.6 cm of patients who accepted surgery alone. However, the difference was not significant (*F* = 1.152, *P* = 0.291).

### *Complications*

Surgical complications were not common in this series. One patient developed local infection after repeated tumor resection and adjuvant radiotherapy. Three patients developed delayed incision healing, recovering after incision debridement and dressing change.

## Discussion

In this study, we presented a relatively large group of patients with primary MLPS in the extremities treated by surgery with or without adjuvant treatments in our hospital. Although the clinical efficacy of chemotherapy based on doxorubicin and ifosfamide and radiotherapy has been reported in the treatment of MLPS^11^, ^12^, ^15^, tumor resection was the first treatment choice in this study. The resection margin is considered one of the most important factors affecting survival in soft tissue sarcoma patients^22^. However, a clinical research of MLPS with 8 years of follow‐up reported that patients who accepted tumor resection with R1 margins had a similar recurrence rate to patients who accepted R0 margins^23^. Another retrospective evaluation of clinical outcomes of liposarcoma patients over 15 years showed no statistically significant relationship between resection margins and patient survival^24^. Wide resection is recommended for soft tissue sarcoma treatment, however, this is not feasible for some tumors with large diameters. In this series, only 11 patients accepted wide resection, while another 23 patients accepted marginal resection. It has been reported that wide resection with limb sacrifice is not necessary for MLPS when the tumor is close to important blood vessels and nerves. The long‐term survival is limited affected even with the potential recurrence risk of liposarcoma^25^. Additionally, it was reported that inadequate surgical margins are a risk factor for local recurrence but not for metastasis^26^. In this study, patients who underwent wide resection had a lower local recurrence rate than patients with marginal resection, but no significant difference was found in the survival analysis. For most patients with large tumor size, marginal resection was the only limb‐sparing option. This result indicates that marginal resection might increase the risk of recurrence but not the risk of death. Otherwise, of the four patients in this study who accepted amputation after MLPS local recurrence, three patients died of the disease.

Although the role of chemotherapy in soft tissue sarcoma treatments remains controversial, adjuvant chemotherapy is recommended in MLPS with large tumor size because of good chemosensitivity to doxorubicin and ifosfamide^12^. Five patients in this series accepted adjuvant chemotherapy consisting of doxorubicin and ifosfamide, of which two patients died of the disease. This result does not deny the efficacy of chemotherapy because patients were chosen nonrandomly for adjuvant treatment based on large tumor size and local recurrent MLPS. Radiotherapy has also been recommended in the treatment of MLPS^15^. In this series, one patient accepted radiotherapy after recrudescence tumor marginal resection, and subsequently became tumor‐free but developed the only case of local infection among the 34 patients. Another patient who accepted radiotherapy for MLPS retroperitoneal metastasis died of the disease without tumor control.

Eight patients (23.5%) developed distant metastases during the study period. Of these, three patients had lung metastases, three patients had bone metastases, one patient had retroperitoneal metastasis, and one patient had multiple metastases. In contrast to other types of liposarcoma or myxoid sarcoma in the extremities, MLPS is associated with an unusual pattern of metastasis. Previous reports showed metastases of MLPS to extra‐pulmonary sites, including the retroperitoneum, bone, and soft tissue^2^, ^27^, ^28^. The bone is a common site for MLPS metastasis, as reported in the literature report^28^. All three patients with bone metastasis in our series showed malignant growths in spinal vertebrae. Localized pain and abnormal signals on MRI suggested MLPS metastasis, although no nuclide concentration was observed on emission CT. The common characteristic of these eight cases with metastases was the large primary tumor size, showing an average tumor diameter of 21.7 cm. This result indicates that tumor size was an independent risk factor for MLPS metastasis.

It is necessary to highlight the limitations of this study. Firstly, because chemotherapy and radiotherapy were used selectively, adjuvant treatment could not be subjected to statistical analysis in this study. Therefore, it is hard to make any definitive statements regarding the use of surgery alone or surgery plus chemotherapy or radiotherapy. However, for most MLPS patients in this series, surgery alone could achieve good tumor control. Secondly, the minimum follow‐up was 12 months, while the longest follow‐up was 238 months. Although all patients accepted treatment in our hospital from 2010 to 2017, patients had different disease stages and some had undergone different operations in the past. Thirdly, tumor location in this study was limited to the extremities and the sarcoma pathological subtype was limited to MLPS. These limitations may lead to incomplete analysis of liposarcoma, but ensure better homogeneity of the patient pool for better comparison.

The optimum treatment of MLPS in the extremities is easily ignored by surgeons, especially in non‐specialized cancer centers. Inappropriate treatments could lead to repeated recurrence of MLPS and negatively impact the outcome of later treatments. Standardized surgery or unplanned secondary operation without delay in a specialized cancer center could benefit patients with MLPS in the extremities. Chemotherapy and radiotherapy may be useful for MLPS, but the results of this study are not definitive. Wide resection could achieve better local control than marginal resection, but there was no significant difference in long‐term survival. Patients with larger MLPS tumors could not undergo wide resection and were more likely to have distant metastases. During the patient follow‐up, unlike with other sarcomas, special attention should be paid to extra‐pulmonary MLPS metastases, including retroperitoneum and bone metastases.
